# Introduction of a second “Green Revolution” mutation into wheat via in planta CRISPR/Cas9 delivery

**DOI:** 10.1093/plphys/kiab570

**Published:** 2021-12-15

**Authors:** Yuya Kumagai, Yuelin Liu, Haruyasu Hamada, Weifeng Luo, Jianghui Zhu, Misa Kuroki, Yozo Nagira, Naoaki Taoka, Etsuko Katoh, Ryozo Imai

**Affiliations:** Genome-Edited Crop Development Group, Institute of Agrobiological Sciences, National Agriculture and Food Research Organization (NARO), Kannondai 3-1-3, Tsukuba, Ibaraki 305-8604, Japan; Genome-Edited Crop Development Group, Institute of Agrobiological Sciences, National Agriculture and Food Research Organization (NARO), Kannondai 3-1-3, Tsukuba, Ibaraki 305-8604, Japan; Biotechnology Research Laboratories, Agri-Bio Research Center, Kaneka Corporation, Iwata, Shizuoka 438-0802, Japan; Genome-Edited Crop Development Group, Institute of Agrobiological Sciences, National Agriculture and Food Research Organization (NARO), Kannondai 3-1-3, Tsukuba, Ibaraki 305-8604, Japan; Genome-Edited Crop Development Group, Institute of Agrobiological Sciences, National Agriculture and Food Research Organization (NARO), Kannondai 3-1-3, Tsukuba, Ibaraki 305-8604, Japan; Genome-Edited Crop Development Group, Institute of Agrobiological Sciences, National Agriculture and Food Research Organization (NARO), Kannondai 3-1-3, Tsukuba, Ibaraki 305-8604, Japan; Biotechnology Research Laboratories, Agri-Bio Research Center, Kaneka Corporation, Iwata, Shizuoka 438-0802, Japan; Biotechnology Research Laboratories, Agri-Bio Research Center, Kaneka Corporation, Iwata, Shizuoka 438-0802, Japan; Structural Biology Team, Advanced Analysis Center, National Agriculture and Food Research Organization, Tsukuba, Ibaraki 305-8602, Japan; Genome-Edited Crop Development Group, Institute of Agrobiological Sciences, National Agriculture and Food Research Organization (NARO), Kannondai 3-1-3, Tsukuba, Ibaraki 305-8604, Japan

## Abstract

Direct delivery of CRISPR/Cas9 ribonucleoproteins into the shoot apical meristem via particle bombardment enabled introduction of a semidwarf1-orthologous mutation into an elite wheat variety.

Dear Editor,

Shoot apical meristems (SAMs) maintain the potential to develop into floral organs. Among the three layers of SAMs, the sub-epidermal layer (L2) is destined to develop into germ cells, such as pollen grains and embryo sacs (Goldberg et al., 1993; Reiser and Fischer, 1993). We developed the in planta particle bombardment (iPB) method for wheat (*Triticum aestivum*) transformation, utilizing SAMs as the target tissue (Hamada et al., 2017; Imai et al., 2020). With this method, genome editing was achieved genotype-independently by transiently expressing clustered regularly interspaced short palindromic repeats (CRISPR)/Cas9 (Hamada et al., 2018; Liu et al., 2021). DNA-free genome-editing systems using direct delivery of CRISPR/Cas9 ribonucleoproteins (RNPs) into plant protoplasts (Woo et al., 2015), fertilized eggs (Toda et al., 2019), or immature embryos (Svitashev et al., 2016; Liang et al., 2017) have been used to create genome-edited plants. These methods, however, require callus culture and regeneration steps which may limit their application strictly to varieties that are amenable to cell/tissue culture. Here, we developed a direct delivery system of Cas9/gRNA RNP into SAMs and established a non-culture method to transform recalcitrant wheat cultivars. 

As shown in Figure 1, a, we delivered gold particles coated with CRISPR/Cas9 RNPs into wheat SAMs (iPB-RNP method) as previously described (Hamada et al., 2017; Imai et al., 2020) and screened E_0_ genome-edited mutants by a cleaved amplified polymorphic sequences (CAPS) assay with fifth leaves. We first used *TaQsd1* (*Tritticum aestivum quantitative trait locus on seed dormancy1*) as a target site and identified five E_0_ positive mutants after two rounds of screening with a CAPS assay (Figure 1, b) which were subsequently validated by Sanger sequencing (Figure 1, c). One plant (Q2) contained mutations in all three homoeologous genes (Figure 1, c). In addition to screening the *TaQsd1* locus, we also deployed this strategy with additional target sites (*Tritticum aestivum Orange* (*TaOr*)_t0, *TaOr*_t1, *Tritticum aestivum hydroxyproline-rich glycoproteins* (*TaHRGP*)-*like1*_t2, Supplemental Table S2) and obtained promising editing efficiency (from 1% to 8.3%) in E_0_ plants (Figure 1, d). Collectively, these results demonstrated that the iPB-RNP method is capable of being deployed for in planta genome editing with comparable efficiency to the iPB-DNA method.

**Figure 1:**
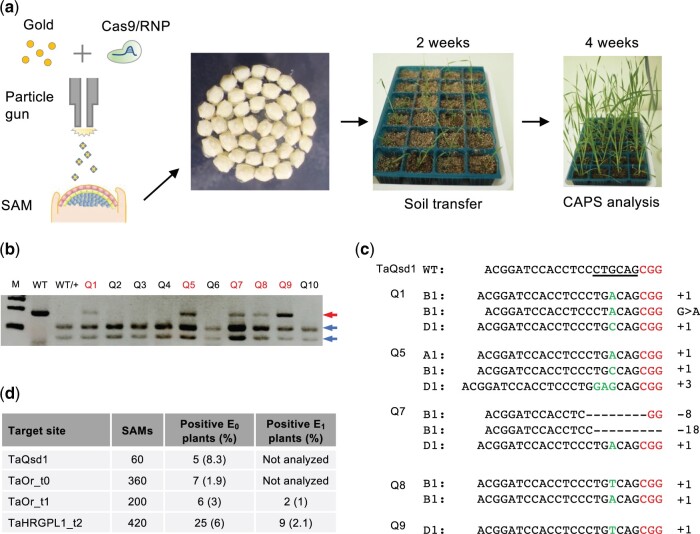
In planta RNP-mediated genome editing in wheat. a, The workflow of the iPB-RNP method utilizing wheat SAMs. b, CAPS analysis of E_0_ plants at the *TaQsd1* locus. The PCR products were amplified by an A, B, and D genome common primer set (Supplemental Table S1). WT, undigested PCR products; WT/+, *Pst* I digested PCR products. Red and blue arrows indicate undigested and digested bands after *Pst* I treatment, respectively. A 100-bp ladder was used as a size marker. c, The genotypes of Q1, Q5, Q7, Q8, and Q9 plants as identified by sequencing. The black and red characters indicate the gRNA and PAM sequences, respectively. The *Pst* I restriction site is underlined. Inserted nucleotides are shown in green characters. d, Summary of genome editing experiment on locus sites of *TaQsd1*, *TaOr*_t0, *TaOr*_t1, and *TaHRGP-like1*_t2 using the iPB-RNP method.

Currently, most commercial wheat cultivars carry a dominant allele of *Reduced height 1* (*Rht1*), a “Green Revolution” gene encoding a GIBBERELLIN-INSENSITIVE (GAI)/DELLA protein (Peng et al., 1999; Hedden, 2003), and have a semidwarf phenotype due to partial gibberellic acid (GA) insensitivity. In contrast, the rice (*Oryza sativa*) semidwarf gene (*sd1*, *semidwarf1*) encodes a GA20 oxidase, which is involved in GA biosynthesis (Monna et al., 2002; Sasaki et al., 2002; Spielmeyer et al., 2002). The impact of the dominant/GA-insensitive and recessive/GA-deficient alleles in wheat and rice, respectively, is affected by their ploidy level. Using genome editing strategies, it is plausible to introduce the recessive *sd1* mutation in *Rht1* wheat and evaluate the effect of the double mutation. With a BLAST search of the Gramene database (http://www.gramene.org), we identified three homoeologous genes, TraesCS3A02G406200, TraesCS3B02G439900, and TraesCS3D02G401400, which encode proteins with 77%–78% identity to rice *sd1* (*OsGA20ox2*). A phylogenetic tree of rice and wheat GA20 oxidases identified four clades, each of which contain one rice and three or four wheat homoeologous genes (Supplemental Figure S1). These results suggest that GA20 oxidases within a clade have an evolutionarily conserved function. Thus, we concluded that *TaSD-A1*, *TaSD-B1*, and *TaSD-D1* were the three wheat orthologs that are homologous to rice *sd1.*

To create a *tasd1* triple knockout mutant using CRISPR/Cas9 RNP, three single-guide RNA (sgRNA) target sequences (target_1, target_2, and target_3) were designed that commonly appear within the *TaSD-A1*, *TaSD-B1*, and *TaSD-D1* genes (Figure 2, a). We evaluated the sgRNA design using an in vitro Cas9 digestion assay. The Cas9 protein in vitro-assembled with the target_2 sgRNA exhibited complete digestion of the target genome sequence under the utilized conditions, while the target_1 and the target_3 sgRNAs were less efficient (Supplemental Figure S2).

**Figure 2:**
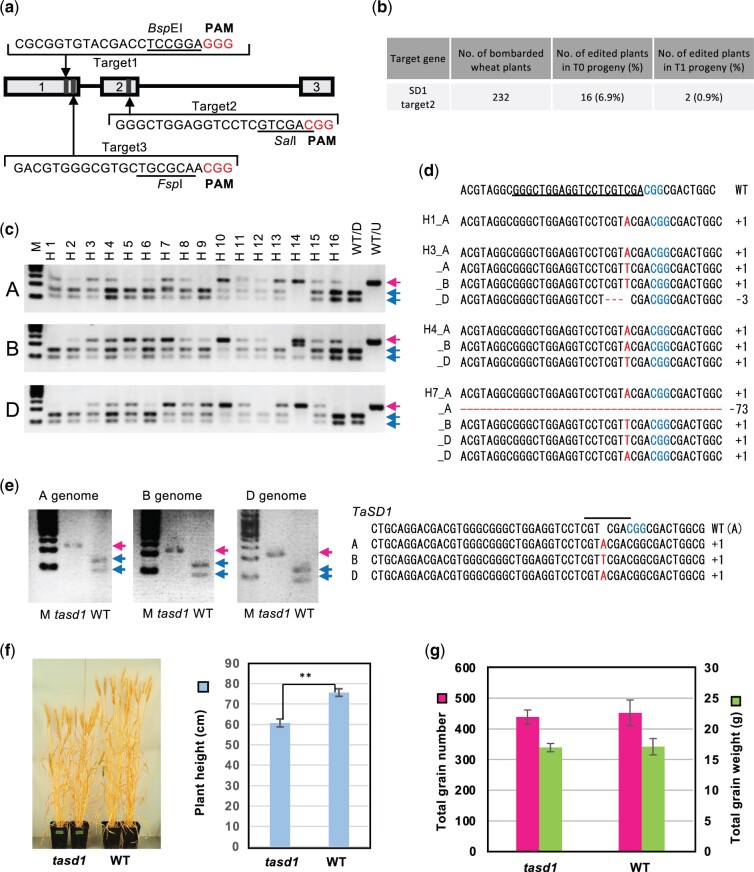
Introduction of *sd1* mutations in wheat. a, Target sequences conserved among the three homoeologous *TaSD1* genes were selected using the CRISPRdirect software. The locations of the target sequences are indicated by arrows. The boxes and lines indicate exons and introns, respectively. The three exons in *TaSD1* are numbered. b, Summary of the CAPS analysis of bombarded E_0_ plants and their progeny. c, CAPS assays of selected positive E_0_ plants using genome-specific primers. WT/D, WT fragment digested with *Sal* I; WT/U, WT fragment undigested. Red and blue arrows indicate undigested and digested bands after *Pst* I treatment, respectively. d, Mutations detected within the target region of positive E_0_ plants. The gRNA sequence is underlined in the WT sequence. Protospacer adjacent motif (PAM) sequences are indicated in blue letters. Insertions and deletions are indicated in red letters. e, A genome-specific CAPS assay of a *tasd1* mutant line (H7-1, E_1_). Red and blue arrows indicate undigested and digested bands after *Sal* I treatment, respectively. The A, B, and D genome sequences of H7-1 are aligned with the A genome sequence of the WT. The inserted nucleotide and PAM sequence are indicated by red and blue letters, respectively. f, Comparison of plant stature of *tasd1* (left) and WT (right) plants. Average tiller height based on measurements of all plants. Data represent the mean ± se of seven *tasd1* and six WT plants. Asterisks indicate statistically significant difference (*t* test, *P* < 0.01). g, Comparison of grain yield. Average total grain numbers and average total grain weight for each plant are shown. The data represent the mean ± se of seven *tasd1* and six WT plants.

Gold particles coated with the CRISPR/Cas9 (target_2) RNP were bombarded into the SAMs of imbibed wheat embryos, prepared as previously described, to enable large-scale screening for *tasd1* mutants (Supplemental Figure S3). We observed undigested bands in 16 plants among the 232 bombarded embryos that had been grown into mature plants, representing 6.9% of the total bombarded embryos (Figure 2, b). A CAPS assay, using genome-specific primers, followed by Sanger sequence analysis of the undigested bands, revealed that the mutations were distributed among the A, B, and D genomes (Figure 2, c and d). Sixteen positive E_0_ plants were subjected to E_1_ genotype analysis. The CAPS assay detected mutant alleles of *tasd1* genes in E_1_ plants derived from two E_0_ plants (H7 and H14, in Figure 2, c and Supplemental Figure S4). Among H7- and H14-derived E_1_ plants, the H7-1 plant did not display a digested band after *Sal* I treatment, suggesting that mutations had occurred in all six *TaSD1* genes (Supplemental Figure S4 and Figure 2, c). The other E_1_ plants displayed digested bands, suggesting WT alleles or partial mutations in the hexaploid genome. A CAPS assay with genome-specific primers indicated that the H7-1 E_1_ plant is a triple mutant (Figure 2, e). Sanger sequencing of the *Sal* I-resistant PCR amplicons revealed that the mutations in the H7-1 plant represent an A, a T, and an A insertion in the A, B, and D genomes, respectively (Figure 2, e). These mutations caused frame shifts that resulted in putative mRNAs with a premature stop codon or no stop codon, suggesting that the TaSD1 function was knocked out (Supplemental Figure S5).

A primer set for *TaSD1* that spans an intron (common to the A, B, and D genomes) was designed and a semi-quantitative RT-qPCR analysis was performed to analyze *TaSD1* expression in the H7-1 E_1_ plants. The expression of the *TaSD1* genes was completely silenced in H7-1 E_1_ plants (Supplemental Figure S6), suggesting the possibility of non-stop or nonsense-mediated mRNA decay.

The phenotype of the *tasd1* mutant was analyzed in the E_3_ generation of the H7-1 line. Both wild-type (WT) and H7-1 mutant plants were grown under long-day conditions in an environmentally controlled growth room. The mutant plants exhibited greener leaf color and shorter plant height. The average final height of the plants was approximately 10% lower in the *tasd1* mutant (Figure 2, f), relative to the WT. The average total number of grains and grain weight was nearly equivalent in WT and *tasd1* plants (Figure 2, g).

We predicted potential off-target sites using Cas-OFFinder and identified 10 candidates having at least two mismatches in the site for target 2. Among them, eight candidates exhibited the same pattern: GGGTTGGAGGTTCTCGTCGAAGG (Underlined bases indicate the mismatches). Therefore, three candidates were selected and five primer sets were designed (Supplemental Table S3). The amplicons produced from the five primer sets were subsequently sequenced and no mutations were found in the potential off-target regions. These data indicate that the mutations occurred without causing any off-target mutations.

In summary, we successfully applied genome editing on different gene loci with the iPB-RNP method utilizing wheat SAMs. We also created a wheat line carrying both *Rht-B1b* and *tasd1* together using genome editing and demonstrated the cumulative effect of the two “Green Revolution” semidwarf genes. The 10% reduction in plant height achieved would further contribute to lodging resistance in current, widely used cultivars. The need for tissue/cell culture in gene-editing techniques hampers the broad utility for a wide range of commercial varieties in many crops, including wheat. The iPB-RNP method described here represents an alternative approach for creating genome-edited wheat varieties with an editing efficiency comparable to the iPB-DNA method, which utilizes transient expression of CRISPR/Cas9 (Hamada et al., 2018). Since no transgene integration occurs when using Cas9 RNPs, the application of the iPB-RNP method in breeding and commercialization has the potential for broad impact to modern agricultural applications.

## Supplemental data

The following materials are available in the online version of this article.

**Supplemental Materials and Methods**.

**Supplemental Figure S1.** Phylogenetic tree of GA20ox from rice and wheat. 

**Supplemental Figure S2.** In vitro Cas9 cleavage analysis.

**Supplemental Figure S3.** CAPS-based screening of *tasd1* mutations using tissue from the 5th leaf of bombarded T0 plants.

**Supplemental Figure S4.** CAPS analysis of T1 plants.

**Supplemental Figure S5.** Semi-quantitative qRT-PCR of *TaSD1* expression in the *tasd1* triple mutant (H7-1).

**Supplemental Figure S6.** Putative amino acid sequences of the mutant TaSD1 proteins.

**Supplemental Table S1.** Sequences of the primers used in this study.

**Supplemental Table S2.** gRNA target sites.

**Supplemental Table S3.** Analysis of possible off-target sites.

## Supplementary Material

kiab570_Supplementary_Data

## Notes

R.I. conceived and supervised the study. Y.K. and R.I. designed the experiments. Y.K., Y.L., W.L., J.Z., and M.K. conducted the experiments. W.L., J.Z., M.K., H.H., Y.N., N.T., and R.I. analyzed the data. Y.K., W.L., and R.I. wrote the manuscript.

The author responsible for distribution of materials integral to the findings presented in this article in accordance with the policy described in the Instructions for Authors (https://academic.oup.com/plphys/pages/General-Instructions) is: Ryozo Imai.
