# Supplementary material for: Introduction of a second “Green Revolution” mutation into wheat via in planta CRISPR/Cas9 delivery
Source: Plant Physiol. 2021 Dec 15;188(4):1838–42. doi: 10.1093/plphys/kiab570 (PMC8968346; doi:10.1093/plphys/kiab570)
Supplement: kiab570_Supplementary_Data [file kiab570_supplementary_data.zip › Supplemental Figures S1S6.pdf]

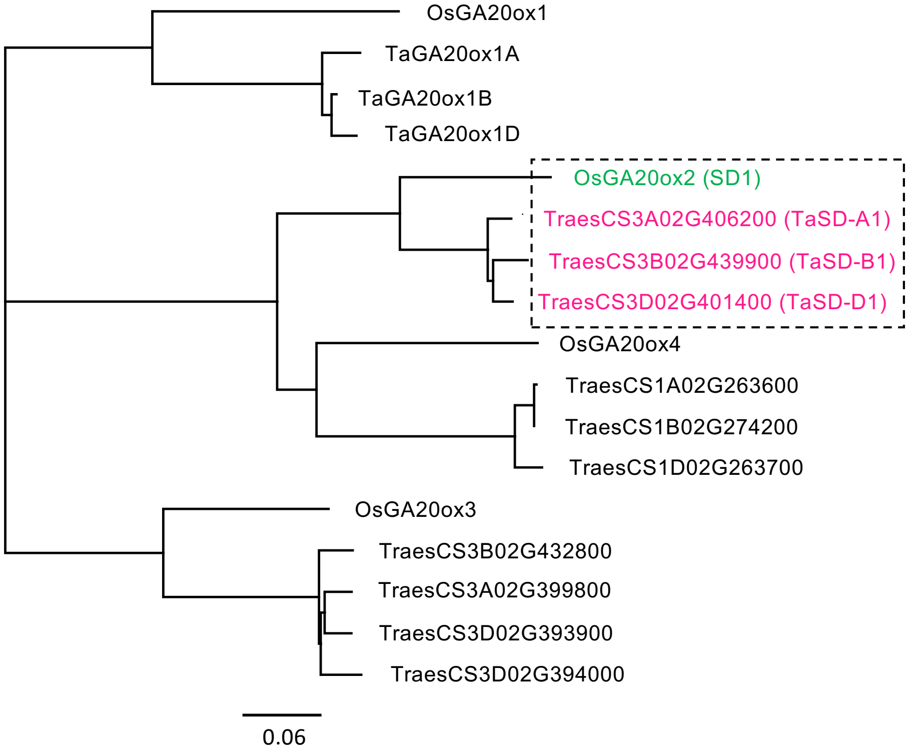

Supplemental Figure S1. Phylogenetic tree of GA20ox from rice and wheat. The amino acid sequences of GA20ox were obtained from the Gramene database (http://www.gramene.org/). A phylogenetic tree was constructed using the neighbor-joining method. The rice SD1 clade is boxed.

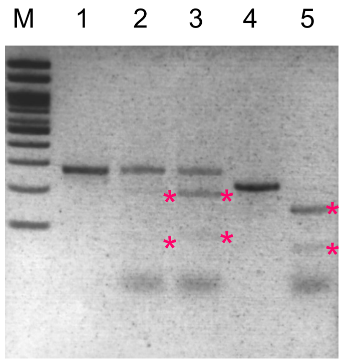

Supplemental Figure S2. In vitro Cas9 cleavage analysis. A genomic fragment containing target 1 and 3 was PCR-amplified (lane 1) and digested with Cas9/gRNAtarget1 (lane 2) and Cas9/gRNAtarget3 (lane 3), respectively. A genomic fragment containing target 2 (lane 4) was also digested with Cas9/gRNAtarget2 (lane 5). Stars denote digested bands.

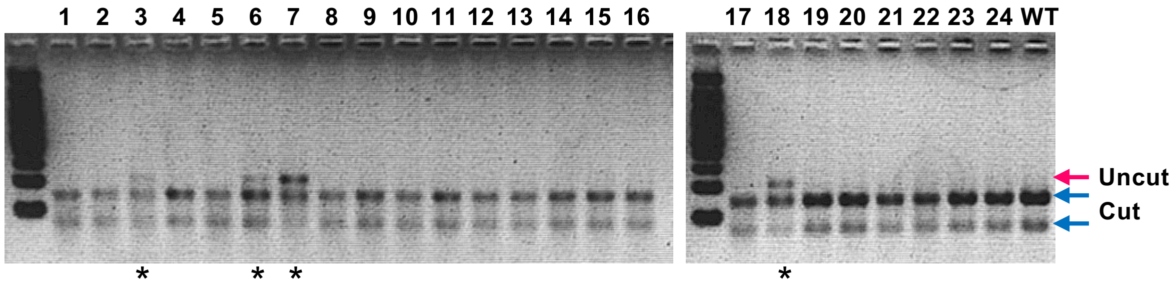

Supplemental Figure S3. CAPS-based screening of sd1 mutations using tissue from the 5th leaf of bombarded T0 plants. A portion of the cleaved, amplified polymorphic sequences (CAPS) assay data for T0 screening. Genomic DNA was isolated from the 5th leaf of the main culm of WT (‘Haruyokoi’) and bombarded T0 plants. A universal primer set (SD1 target 2F and SD1 target 2R) was used to amplify all three homoeologous genes. Red and blue arrows indicate undigested and digested bands after *Sal* I treatment, respectively. Stars denote samples displaying positive signals.

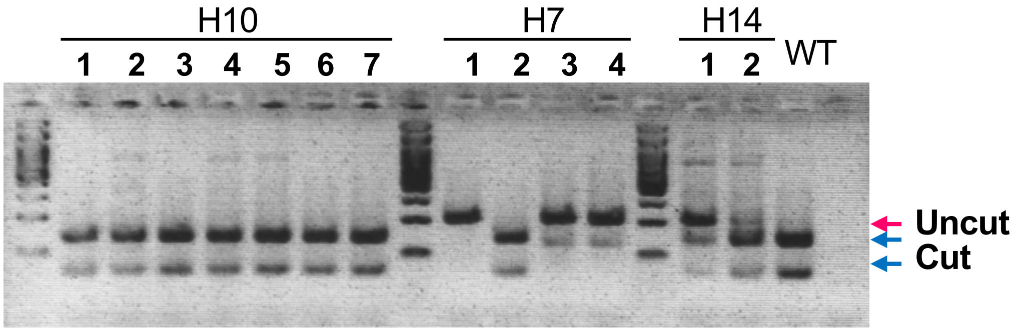

Supplemental Figure S4. **CAPS analysis of T1 plants**. Leaf tissue of T_1 plants from T_0 positive plants (H7, H10, H14) was utilized to conduct a CAPS analysis with *Sal* I digestion. Due to dense planting in small pots, only seven (H7), four (H10), and two (H14) E1 seeds were obtained, respectively. Genomic DNA was isolated from the 1st leaf of WT and the T_1 plants. A universal primer set (SD1 target 2F and SD1 target 2R) was used to amplify all three homoeologous genes. Red and blue arrows indicate undigested and digested bands after *Sal* I treatment, respectively.

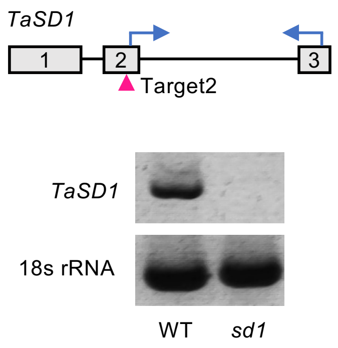

Supplemental Figure S5. **Semi-quantitative qRT-PCR of SD1 expression in the *sd1* triple mutant (H7-1)**. Primer locations are indicated by arrows. Targeted mutation site is indicated by red arrowhead. semi quantitative RT-PCR was carried out with leaf tissue of T1 plants.

WT-A: MDTSPATPLLLQPPAPSIDPFAAKAAVNKNGGAATAVYDLRREPKIPAPFVWPHAEVRPTTAEELAVPVVDVGVLRNGDA
H7-1_A: MDTSPATPLLLQPPAPSIDPFAAKAAVNKNGGAATAVYDLRREPKIPAPFVWPHAEVRPTTAEELAVPVVDVGVLRNGDA

AGLRRAVAQVAAACATHGFFQVSGHGVDDALARAALDGASGFFGLPLAEKQRARRVPGTVSGYTSAHADRFASKLPWKET
AGLRRAVAQVAAACATHGFFQVSGHGVDDALARAALDGASGFFGLPLAEKQRARRVPGTVSGYTSAHADRFASKLPWKET

LSFGFHDRAGAAPVVVDYFTSTLGPDYEPMGRVYQEYCEKMKELSLRIMELLELGLGVEKRGYYRDFFADSSSIMRCNYY
LSFGFHDRAGAAPVVVDYFTSTLGPDYEPMGRVYQEYCEKMKELSLRIMELLELGLGVEKRGYYRDFFADSSSIMRCNYY

PPCPEPERTLGTGPHCDPTALTILLQDDVGGLEVLVDGDWRPVRPVPGAMVINIGDTFMALSNGRYKSCLHRAVVNRRQE
PPCPEPERTLGTGPHCDPTALTILLQDDVGGLEVLVRRRLAARPPRPRRHGHQHRRHLHGAVERAVQELPAPGGGEPAAG

RRSLAFFLCPREDRVVRPPPGLRSPRRYPDFTWADLMRFTQRHYRADTRTLDAFTQWFSSSTSPPPPAPAAQQAA*
AAVAGLLPVPARGPRGAAAAGPEEPAAVPGLHLGRPHALHAAPLPRRHAHPRRLHPVVLLLHLAAAARPGGPAGGLIASP

DPIDPRADSPRGSRRGIFVGTSPRARAPPSQVRWARAEWCPRGFPAPHHLPFLDAGSRSLLLALFVTTRHMHA

WT-B: MVLQTAQQEPSLTRPPHCSAASARSPAAMDTSPATPLLLQPPAPSIDPFAAKAAVNKNGGAATAVYDLRREPKIPAPFVW
H7-1_B: MVLQTAQQEPSLTRPPHCSVASARSPAAMDTSPTTPLLLQPPAPSIDPFAAKAAVNKNGGAATAVYDLRREPKIPAPFVW

PHAEVRPTTAEELAVPVVDVGVLRNGDAAGLRRAVAQVAAACATHGFFQVSGHGVDDALARAALDGASGFFGLPLAEKQR
PHAEVRPTTAEELAVPVVDVGVLRNGDAAGLRRAVAQVAAACATHGFFQVSGHGVDDALARAALDGASGFFGLPLAEKQR

ARRVPGTVSGYTSAHADRFASKLPWKETLSFGFHDRAGAAPVVVDYFTSTLGPDYEPMGRVYQEYCEKMKELSLRIMELL
ARRVPGTVSGYTSAHADRFASKLPWKETLSFGFHDRAGAAPVVVDYFTSTLGPDYEPMGRVYQEYCEKMKELSLRIMELL

ELGLGVEKRGYYRDFFADSSSIMRCNYYPPCPEPERTLGTGPHCDPTALTILLQDDVGGLEVLVDGDWRPVRPVPGAMVI
ELGLGVEKRGYYRDFFADSSSIMRCNYYPPCPEPERTLGTGPHCDPTALTILLQDDVGGLEVLVRRRLAPRPPRPRRHGH

NIGDTFMALSNGRYKSCLHRAVVNRRQERRSLAFFLCPREDRVVRPPPGLRSPRRYPDFTWADLMRFTQRHYRADTRTLD
QHRRHLHGSVERAVQELPAPRGGEPAAGAAVAGLLPVPARGPRGAAAAGAEEPAAVPGLHLGRPHALHAAPLPRRHAHPR

AFTQWFSSSTSPPPPAPAAQQAA*
RLHPVVLLLLLLLLLLGPGGGLILLLPIDPRADSTRGSRHEFLSGPAHVRAPPFSGAVARRGVVPTWISGPTPPSIFGRW

LASPPPSLVCHDSPYACPLL

WT-D: MDTSPATPLLLQPPAPSIDPFAAKAAVNKGGGAATAVYDLRREPKIPAPFVWPHAEVRPTTAQELAVPVVDVGVLRNGDA
H7-1_D: MDTSPATPLLLQPPAPSIDPFAAKAAVNKGGGAATAVYDLRREPKIPAPFVWPHAEVRPTTAQELAVPVVDVGVLRNGDA

AGLRRAVAQVAAACATHGFFQVSGHGVDEALARAALDGASGFFRLPLAEKQRARRVPGTVSGYTSAHADRFASKLPWKET
AGLRRAVAQVAAACATHGFFQVSGHGVDEALARAALDGASGFFRLPLAEKQRARRVPGTVSGYTSAHADRFASKLPWKET

LSFGFHDRAGAAPVVVDYFTSTLGPDYEPMGRVYQEYCGKMKELSLRIMELLELSQGVEKRGYYREFFADSSSIMRCNYY
LSFGFHDRAGAAPVVVDYFTSTLGPDYEPMGRVYQEYCGKMKELSLRIMELLELSQGVEKRGYYREFFADSSSIMRCNYY

PPCPEPERTLGTGPHCDPTALTILLQDDVGGLEVLVDGDWRPVRPVPGAMVINIGDTFMALSNGRYKSCLHRAVVNRRQE
PPCPEPERTLGTGPHCDPTALTILLQDDVGGLEVLVRRRLAPRPPRPRRHGHQHRRHLHGNYSSLSVAFAD*

RRSLAFFLCPREDRVVRPPPGLRSPRRYPDFTWADLMRFTQRHYRADTRTLDAFTQWFSSSSSSAQEAA*

Supplemental Figure S6. Putative amino acid sequences of the mutant TaSD1 proteins.
The upper amino acid sequence is the wild type and the lower is the genome-edited amino acid sequence of TaSD1. Altered amino acids are indicated by red characters.
