# Supplementary material for: Introduction of a second “Green Revolution” mutation into wheat via in planta CRISPR/Cas9 delivery
Source: Plant Physiol. 2021 Dec 15;188(4):1838–42. doi: 10.1093/plphys/kiab570 (PMC8968346; doi:10.1093/plphys/kiab570)
Supplement: kiab570_Supplementary_Data [file kiab570_supplementary_data.zip › Supplemental Materials and Methods.edited.pdf]

Supplemental Materials and Methods

Preparation of SAMs. The protocol for SAM preparation has been previously described[1]. In brief, mature seeds of wheat (*Triticum aestivum*) 'Haruyokoi' (*RhtB1-b*, *RhtD1-a*) were sterilized with sodium hypochlorite and imbibed at 25˚C overnight. The coleoptile and the first three leaves, which cover the SAM, were removed from the embryo under a stereo microscope using an insulin pen needle 34G (ϕ0.2 mm; TERUMO, Japan). The embryos were separated from the endosperm and placed upright in a petri dish containing Murashige and Skoog (MS) basal medium supplemented with maltose (30 g/L), 2-morpholinoethanesulfonic acid (MES) monohydrate (0.98 g/L, pH 5.8), a plant preservative mixture (3%; Nacalai Tesque, Japan), and phytagel (7.0 g/L; Sigma Aldrich, USA). Thirty embryos were placed on the medium in each petri dish for each cycle of particle bombardment.

Preparation of Cas9 protein and sgRNA. Recombinant *Streptococcus pyogenes* Cas9 protein was purified from *Escherichia coli* as previously described[2]. Single-stranded, guide RNA (sgRNA) was prepared using a GeneArt™ Precision gRNA synthesis Kit (Thermo Fisher Scientific, USA). The templates for the *in vitro* transcription were designed and amplified using appropriate primers (Supplemental Table S1) according to the manufacturer's instructions.

In vitro **digestion of CRISPR/Cas9 RNP.** DNA fragments containing the target sites were amplified from genomic wheat DNA using designated primer sets (Supplemental Table S1), purified and dissolved in RNase-free water. Cas9 protein (0.2 µg) and sgRNA

(0.2 μg) were mixed and left for 10 min at room temperature to form an RNP complex. The RNP was incubated with the purified target DNA (100–200 ng) in CutSmart® buffer (New England BioLabs) in a total volume of 10 μL, and digestion was allowed to proceed for 1 h at 37°C. The digested products were then separated on a 3% agarose gel.

Preparation of microprojectiles and biolistic delivery. Gold particles coated with Cas9 RNP were prepared as previously described[3] with slight modification. The purified Cas9 protein (12 μg) and sgRNA (5 μg) were mixed in a binding buffer (20 μL) containing 5 μL of 10×CutSmart® buffer and 1μL of RNase inhibitor (40U, Takara, Japan) and left for 10 min at room temperature. After addition of 5 μL of TransIt transfection reagent (TaKaRa), the mixture was allowed to sit for an additional 5 min. Two hundred and seventy micrograms of gold particles (0.6 μm, InBio Gold, Australia) were added to the RNP mixture, tap-mixed, and then left to sit for 10 min. The gold particles were subsequently dispersed by slight sonication and 5 μL of the mixture was loaded onto a hydrophilic film (Scotchtint, 3M, Japan), placed on a macrocarrier and allowed to air-dry at room temperature for 15 min. Bombardment was conducted using a PDS-1000/He™ device (Bio-Rad, USA) with a target distance of 6.0 cm from the stopping plate. The vacuum in the chamber was 27 inches of Hg and the helium pressure was 1350 psi. Bombardment was repeated three times per plate.

Cleaved amplified polymorphic sequences (CAPS) analysis. Genomic DNA was extracted from the fifth leaf of E_0 progeny and the first leaf of the E_1 progeny as previously described[4] For *TaOr* (AK457010.1) and *HPGP-like* (AK333546.1), PCR amplification was conducted using KOD FX Neo DNA polymerase (Toyobo, Osaka, Japan) with gene

specific primers (300 nM of each), and genomic DNA (50 ng). The mixture was denatured for 2 min at 98°C in a thermocycler and then subjected to 30 cycles of amplification (98°C for 30 s, 60°C for 30 s, 68°C for 30 s). For *TaQsd1* (LC209619.1) and *TaSD1*, PCR amplification was conducted using TaKaRa LA Taq® with GC buffer (TaKaRa), gene specific primers (300 nM of each), and genomic DNA (50 ng). The mixture was denatured for 2 min at 94°C in a thermocycler and then subjected to 30 cycles of amplification (94°C for 30 s, 55°C for 30 s, 72°C for 20 s). The common primer and genome-specific primer sets used in the PCR are listed in Supplementary Table 1. The amplified PCR products were digested with *Pst* I (TaQsd1), *Nde* I (HRGP-like1_t2), *Sal* I (TaSD1_t2) or Cas9 RNPs (TaOr_t0 and TaOr_t1) and subsequently analyzed by agarose gel electrophoresis. Undigested bands from the restriction enzyme digestion were purified and cloned into the pGEM-T easy vector (Promega, USA) or using Zero Blunt™ TOPO™ PCR Cloning Kit (Invitrogen™, USA), and sequenced on a 3130xl Genetic analyzer (Applied Biosystems, USA).

Plant growth conditions. Twelve hours after bombardment, the embryos were transferred to a basal MS medium and cultured for 2–3 weeks in a growth chamber under long day conditions (16 h light/8 h darkness, 25°C). The seedlings were subsequently planted in pots (3 seedlings/pot, ϕ10.5 cm) and grown in a phytotron under long day conditions (16 h light/8 h darkness, 20°C).

RT-qPCR analysis. Total RNA was isolated from leaf tissue using a RNeasy Mini Kit (Qiagen, Hilden, Germany) according to the manufacturer's protocol. First-strand cDNA was synthesized from total RNA (0.5 μg) using a PrimeScript™ II 1st strand cDNA

Synthesis Kit (TaKaRa, Japan). PCR was conducted with TaKaRa LA Taq® with GC buffer (TaKaRa) as follows: initial denaturation (94°C for 1 min), followed by 28 cycles (98°C for 10 s, 55°C for 15 s, and 72°C for 30 s) using specific primers for 18s rRNA and *TaSD1* (Supplemental Table S1).

Phylogenetic tree. The amino acid sequences of GA20ox proteins were obtained from the Gramene database (http://www.gramene.org/). A phylogenetic tree was constructed using the neighbor-joining method. Bootstrap values were calculated from 1000 replicates.

Off-target detection. PCR analysis was conducted to detect off-target mutagenesis in E_1 mutants. Off-target sites were identified by Cas-OFFinder (http://www.rgenome.net/cas-offinder/)[4]. DNA was isolated from the first leaf of E_1 plants, as previously described[1]. Each PCR was conducted using TaKaRa LA Taq® with GC buffer (TaKaRa), according to the manufacturer's instructions, along with the designated primers (300 nM of each: Supplemental Table S1) and genomic DNA (50 ng). The mixture was denatured for 2 min at 94°C in a thermocycler and then subjected to 30 cycles of amplification (94°C for 30 s, 55°C for 30 s, 72°C for 30 s). The resulting PCR products were then sequenced and analyzed.

Sequencing analysis. PCR products used in the CAPS analysis and off-target detection were cloned into pCR-BluntII-TOPO (Thermo Fisher Scientific, USA) and sequenced on a 3130xL genetic analyzer (Applied Biosystems, USA).

Data Availability. All data generated or analyzed during this study are included in this published article (and its Supplementary Information files). Regarding sequence data, the NCBI GenBank identifiers are: LC209619.1 (*TaQsd1*), AK457010.1(*TaOr*), AK333546.1 (*TaHRGP-like*) and LN828667.1 (*TaSD1*).

References

1. Hamada, H. *et al.* An in planta biolistic method for stable wheat transformation. *Sci. Rep.* **7**, 11443 (2017).

2. Svitashev, S., Schwartz, C., Lenderts, B., Young, J. K. & Mark Cigan, A. Genome editing in maize directed by CRISPR–Cas9 ribonucleoprotein complexes. *Nat. Commun.* **7**, 13274 (2016).

3. Kunitake, E. *et al.* CRISPR/Cas9-mediated gene replacement in the basidiomycetous yeast Pseudozyma antarctica. *Fungal Genet. Biol.* **130**, 82–90 (2019).

4. Bae, S., Park, J. & Kim, J. S. Cas-OFFinder: A fast and versatile algorithm that searches for potential off-target sites of Cas9 RNA-guided endonucleases. *Bioinformatics* **30**, 1473–1475 (2014).
