# Supplementary material for: Introduction of a second “Green Revolution” mutation into wheat via in planta CRISPR/Cas9 delivery
Source: Plant Physiol. 2021 Dec 15;188(4):1838–42. doi: 10.1093/plphys/kiab570 (PMC8968346; doi:10.1093/plphys/kiab570)
Supplement: kiab570_Supplementary_Data [file kiab570_supplementary_data.zip › SuppTablesS1S3.pdf]

Supplemental Table S1. Sequences of the primers used in this study.

Primer name	Primer sequence (5'-3')	Target	Purpose
TaQsd1 F	CAGCCTGGAGGGAATGACC	A, B and D genome	To amplify the TaQsd1 genome region target site
TaQsd1 R	ACCTGGTGGAATCCAGAGC		
TaQsd1 gRNA F	TAATACGACTCACTATAGACGGATCCACCTCCCTG	TaQsd1	Template DNA amplification for gRNA synthesis
TaQsd1 gRNA R	TTCTAGCTCTAAAACCTGCAGGGAGGTGGATCCGT		
TaOr F	ACATCCATGGATCCTTTAGGATTAG	A, B and D genome	To amplify the TaOr genome region target (t0 and t1) sites
TaOr R	GCATAGTAGACCTTCAGATTAGCTG		
TaOr t0 gRNA F	TAATACGACTCACTATAGGTAAGGGCCTACTAACC	TaOr_t0	Template DNA amplification for gRNA synthesis
TaOr t0 gRNA R	TTCTAGCTCTAAAACCTGGTTAGTAGGCCCTTACC		
TaOr t1 gRNA F	TAATACGACTCACTATAGTGGTAAGGGCCTACTAA	TaOr_t1	Template DNA amplification for gRNA synthesis
TaOr t1 gRNA R	TTCTAGCTCTAAAACTGGTTAGTAGGCCCTTACCA		
TaHRGPL1 F	AAGCTTCTGACTTCACTCAACAG	A, B and D genome	To amplify the TaHRGPL1 genome region target site
TaHRGPL1 R	TGAGGAAGTCTGCAACTCAG		
TaHRGPL1 t2 gRNA F	TAATACGACTCACTATAGTTGGGCTCTCAGGGTAC	TaHRGPL1_t2	Template DNA amplification for gRNA synthesis
TaHRGPL1 t2 gRNA R	TTCTAGCTCTAAAACTATGTACCCTGAGAGCCCAA		
SD1 F	AGATGAAGGAGCTGTCGCTG	A and D genomes	To amplify the TaSD1 genome region target 1-3 sites
SD1 R	GGAAGCAGAGTGCAATCACG		
SD1 F2	GATCATGGAGCTGCTGGAGC	B genome	To amplify the TaSD1 target 1-3 sites
SD1 R2	TGAAGGTGTCGCCGATGTTG		
SD1 target 1:3 F	TCCCAGCATTGACCCGTTCG	Target 1 and 3	To amplify the TaSD1 target1 and target3 sites for CAPS
SD1 target 1:3 R	ACACCTGGAAGAACCCGTGC		
SD1 target 2 F	CGGACAGCAGCTCCATCATG	Target 2	To amplify the TaSD1 target2 site for CAPS
SD1 target 2 R	GGTGTCGCCGATGTTGATGA		
SD1 target 2A R	CTGATACGAGCAGCAGTAGC	Target 2A	Genome-specific primers for the TaSD1 target2 site amplification with the SD1 target 2 F
SD1 target 2B R	GCAAGGAAGGTGACCCAATT	Target 2B	
SD1 target 2D R	AGCAACGCTGAGAGAGGAGT	Target 2D	
gRNA target 1 F	TAATACGACTCACTATAGCGCGGTGTACGACCTCCGGA	Target 1	Template DNA amplification for gRNA synthesis
gRNA target 1 R	TTCTAGCTCTAAAACTCCGGAGGTCGTACACCGCG		
gRNA target 2 F	TAATACGACTCACTATAGGGCTGGAGGTCCTCGTCGA	Target 2	Template DNA amplification for gRNA synthesis
gRNA target 2 R	TTCTAGCTCTAAAACTCGACGAGGACCTCCAGCC		
gRNA target 3 F	TAATACGACTCACTATAGACGTGGGCGTGCTGCGCAA	Target 3	Template DNA amplification for gRNA synthesis
gRNA target 3 R	TTCTAGCTCTAAAACTTGCGCAGCACGCCCACGT		
SD1 RTPCR R	GCACGTGGGCTGGTCCCGACA	TaSD1	RT-PCR
SD1 RTPCR F	TCATCAACATCGGCGACACC		
Ta18s RTPCR F	GTGACGGGTGACGGAGAATT	18s rRNA	RT-PCR
Ta18s RTPCR R	GACACTAATGCGCCCGGTAT		
Off target 1 F	TCCCGCCTTTCTCTGAATG	1026	Off target detection
Off target 1 R	CCACCTTCACCAACACTTTC		
Off target 2 F	TGCGCGATTCATCGGCCAAC	1578	Off target detection
Off target 2 R	AGTCTGGTGAACACCGCCAT		
Off target 3 F	TTCCTAAGGACATTTGTGAGGTTA	1642	Off target detection
Off target 3 R	TCGTGGACATTGCTGCAACC		
Off target 4 F	ACCAGCACCTTCTCCAGAAC	1032	Off target detection
Off target 4 R	CCTTGACGGAGACATTGGAC		
Off target 5 F	CCCAGAATCTTCTCAGGGATAC	2681	Off target detection
Off target 5 R	ACTAGCACCTTCTCCAGAAC		

Supplemental Table S2. gRNA target sites.

gRNA	Target (5'-3')	NCBI accession ID
TaQsd1	ACGGATCCACCTCCCTGCAGCGG	LC209619.1
TaOr_t0	GGTAAGGGCCTACTAACCAGGGG	AK457010.1
TaOr_t1	TGGTAAGGGCCTACTAACCAGGG	AK457010.1
TaHRGPL1_t2	TTGGGCTCTCAGGGTACATATGG	AK333546.1
TaSD1_t1	CGCGGTGTACGACCTCCGGAGGG	LN828667.1
TaSD1_t2	GGGCTGGAGGTCCTCGTCGACGG	LN828667.1
TaSD1_t3	GACGTGGGCGTGCTGCGCAACGG	LN828667.1

The PAM motif in each target sequence is shown in red.

Supplemental Table S3. Analysis of possible off-target sites.

#	Target	Chromosome Location	Position	Mismatch	Mutation
1	GGGCTGGAGGTtCTCGTCGgAGG	TGACv1_scaffold_014114_1AL	1026	2	–
2	GGGCTGGAGGTtgTCGTCGAAGG	TGACv1_scaffold_386600_5AL	1578	2	–
3	GGGtTGGAGGTtCTCGTCGAAGG	TGACv1_scaffold_117732_2AS	1642	2	–
4	GGGtTGGAGGTtCTCGTCGAAGG	TGACv1_scaffold_206147_3AL	1032	2	–
5	GGGtTGGAGGTtCTCGTCGAAGG	TGACv1_scaffold_660168_U	2681	2	–
6	GGGtTGGAGGTtCTCGTCGAAGG	TGACv1_scaffold_007802_1AL	2718	2	N.D.
7	GGGtTGGAGGTtCTCGTCGAAGG	TGACv1_scaffold_200959_3AL	3374	2	N.D.
8	GGGtTGGAGGTtCTCGTCGAAGG	TGACv1_scaffold_654903_U	3265	2	N.D.
9	GGGtTGGAGGTtCTCGTCGAAGG	TGACv1_scaffold_215894_3AS	2609	2	N.D.
10	GGGtTGGAGGTtCTCGTCGAAGG	TGACv1_scaffold_680557_U	304	2	N.D.

N.D: Not determined. Red letters indicate mismatches
